# Supplementary material for: Two casting methods compared in patients with Colles' fracture: A pragmatic, randomized controlled trial
Source: PLoS One. 2020 May 29;15(5):e0232153. doi: 10.1371/journal.pone.0232153 (PMC7259650; doi:10.1371/journal.pone.0232153)
Supplement: S1 Appendix — (DOCX) [file pone.0232153.s002.docx]

Appendix – Inclusion and exclusion criteria of the study

**Inclusion and exclusion criteria**

No changes were made to the inclusion and the exclusion criteria:Inclusion criteria

- Low energy intra- or extra-articular dorsal primarily stable, reducible DRF within 3 cm of the radiocarpal joint diagnosed with lateral and posterior-anterior radiographs in ER
- Physician on call (general practitioner, acute physician, orthopaedic resident, orthopaedic consultant) thinks patient would be suitable for non-operative treatment

Exclusion criteria

- Operative treatment
- Refused to participate in the study
- Open fracture more than Gustilo 1 gradus
- Under 65 years of age
- Chauffeure’s or Barton’s fracture
- Smith’s fracture (volar angulation of the fracture)
- Does not understand written and spoken guidance in local languages
- Pathological fracture or previous fracture in the same wrist, forearm, or elbow
