# Supplementary material for: Two casting methods compared in patients with Colles' fracture: A pragmatic, randomized controlled trial
Source: PLoS One. 2020 May 29;15(5):e0232153. doi: 10.1371/journal.pone.0232153 (PMC7259650; doi:10.1371/journal.pone.0232153)
Supplement: S2 Appendix — (DOCX) [file pone.0232153.s003.docx]

Appendix – Changes in Methods and Design of the Protocol

During the trial, a few changes occurred in the Methods from those described in the original protocol. These changes are explained in more detail below.

**Methods and Design**

**Follow-up and outcome assessment**

The primary outcome, PRWE at 1- and 2-year follow up was assessed by sending the patients all the paper forms that needed to be filled out for the patient-rated outcome measures, including PRWE, by surface mail instead of using ”an Internet based system” which had originally been stated in the protocol.

In the protocol, it was planned that the range of motion of the fractured wrist would be assessed at the 3-month outpatient clinic visit. However, one of the three research centers did not systematically collect this data, and thus the range of motion was not included in our study results.

**Power analysis**

In the protocol, the calculated sample size for the analysis was 40 patients per group adjusted to a drop-out rate of 30%, giving 57 (40 divided by 0.7) patients per group (114 in total). However, we only recruited 105 patients due to a smaller drop-out rate than anticipated. At 12 months, we had valid answers from 86 recruited patients in the primary outcome measure, PRWE at 12 months.

**Statistical analysis**

In the protocol, we stated that the chi-square test would be used for dichotomous variables which were analysed with Fisher’s exact test due to the better fit for small sample size.

With regard to subgroup analysis, we stated the following in the protocol: ”In subgroup analysis, the effect of age, sex, fracture group, smoking and other diseases will be evaluated against the scores and overall quality of life after fracture.” However, our data on smoking status and other diseases were judged not to be valid enough to be incorporated in the results, and thus for subgroup analysis. Instead, in the subgroup analysis, we utilised age, sex, handedness, PCS, weekly distance span, ancillary use outside of home, which were assumed to be the best estimates available in our study data to predict the 12-month results in the primary outcome.

In addition to the study protocol, we tested an additional fourth hypothesis (does the grip strength in the contralateral side of the fracture correlate with the PRWE measure), the pearson’s correlation of the 15D (health-related quality of life) measure to PRWE measure.

**The trial registration**

The trial was registered with Clinicaltrials.gov two months after the first recruitment to the study. A total of three patients were recruited before the registration at Tampere University Hospital, the main study center.
