# Supplementary material for: Two casting methods compared in patients with Colles' fracture: A pragmatic, randomized controlled trial
Source: PLoS One. 2020 May 29;15(5):e0232153. doi: 10.1371/journal.pone.0232153 (PMC7259650; doi:10.1371/journal.pone.0232153)

Appendix – Additional analyses

In this appendix, we describe those analyses that were not stated in the published protocol but were reported in the article. Furthermore, we explain in more detail the rationale behind these analyses.

**Multiple imputation for missing data**

For missing data on PRWE, qDASH, VAS for pain, PCS, and 15D, we performed multiple imputation using the mice() function in the R software. In the linear regression model, the variables were age, sex, cast position (treatment allocation), the PRWE at 3 and 12 months, the PCS at 3 and 12 months, the VAS for pain at 3 and 12 months, and the Quick-DASH at 3 and 12 months of follow-up. The multiple imputation was repeated 5 times for each missing value. Each data set was analyzed for the variable parameters and these were then averaged for a single estimate. The Student’s t-test was used to compare the differences in means between the VFUDC and FC groups adjusted for the missing data.

**PROMs and Health-related quality of life**

The primary outcome, PRWE at 12 months, was stated in the results section of the study with the multiple imputation method and without multiple imputation. We reported the results of the multiple imputation as follows:

”After adjustment by patients lost to follow-up using the multiple imputation method, between group differences in means (CI) at 12 months for PRWE score was -3.9 (-12.0 – 4.2, p=.34).”

The result in between-group differences in PRWE without multiple imputation was reported as follows:

”At 12 months, the mean (CI for difference in means) PRWE score was 15.5 and 20.4 (-13.1 – 3.4, p=.24)”

In addition to the fourth hypothesis of the protocol (does the grip strength of the contralateral side of the fracture as a marker of the general physical ability correlate to the PRWE), a correlation test betweeen the 15D and PRWE as an association test between the general health and the PRWE measure was performed. We reported this as follows:

”The correlation between 15D and PRWE was present at 3 months (CC: -0.41, p<.001) and at 12 months (CC: -0.41, p<.001).”

**Radiographs**

The internal validation of the casting procedure was assessed by radiographs after the closed reduction. This assessment was not stated in the protocol. We reported this as follows:

”The radiographic confirmation of the differences between the two immobilization methods was performed using measurements of ulnar deviation of the third metacarpal compared with the radial axis and angulation of the wrist comparing second metacarpal flexion/extension to radial axis, having differences in means (CI) -4.0 (-6.0 - -2.0, p<.001) and 12.2 (8.2-16.2, p<.001) degrees of difference between the groups, respectively.”

**PCS**

Our hypothesis in the protocol was the presence of the correlation between the PCS at the baseline and the PRWE at 12 months. There was, however, no correlation between these measures. The catastrophize thinking of pain may emerge after experiencing painful conditions according to the theory that the PCS score is based on. Therefore, we assessed the correlation between the change in the patient-specific PCS score between the baseline and 12 months of follow-up to the PRWE at 12 months. We reported this as follows:

”An increase in the patient-specific PCS value between baseline and 12 months did correlate moderately with a worsening (increase in value) of the PRWE score at 12 months (CC: 0.24, p=.05).”

**Complications:**

The statistical significance of the number of cast changes between intervention groups was tested as stated in the protocol (Chi’s square test). In addition, we tested the association of the cast changes to the PRWE measure at 12 months by Student’s t-test, in accordance with the hypothesis that people who have experienced cast change may have less courage to rehabilitate the wrist and hand. Due to the small number of patients who had 2 or more cast changes, we divided the patients into two groups: those who had one or more cast change(s) and those who had no cast change during the 5-week cast immoblization period. We reported this test as follows:

” In addition, the mean difference in PRWE score at 12 months was -8.5 points (CI: -18.0 – 1.1, p=.08) lower if no cast changes occurred compared with one or more cast changes.”

**Extra- and intra-articular fractures**

In the randomization process, we stratified the recruited patients in 2x2 blocks to the treatment groups. The blocks were age (under and over 75 years of age) and extra- or intra-articular fracture line due to the hypothesis that intra-articular freactures have a worse prognosis.

Table 1. Mean differences in the primary and the secondary outcomes compared between extra- and intra-articular fractures

| Measure | Extra (N) | Intra (N) | Extra, mean | Intra, mean | Mean difference | 95% CI | p-value |
| --- | --- | --- | --- | --- | --- | --- | --- |
| PRWE 12m | 65 | 21 | 16.8 | 22.5 | -5.8 | -15.3 – 3.7 | 0.23 |
| qDASH 12m | 63 | 21 | 16.9 | 24.2 | -7.3 | -16.0 – 1.3 | 0.10 |
| VAS for pain 12m | 64 | 20 | 13.9 | 15.5 | -1.6 | -10.9 – 7.6 | 0.35 |
| Grip strength, proportion, 3m | 64 | 28 | 0.49 | 0.45 | 0.05 | -0.04 – 0.14 | 0.31 |

The Pearson’s correlation coefficients between various PROMs of the study at 3 and 12 months of follow-up (Table 2).

Table 2. Pearson’s correlation coefficients between various PROMs of the study at 3 and 12 months of follow-up

| Measure | | PCS baseline | PCS 3m | PCS change 0-12m | PRWE 12m | qDASH 12m | VAS for pain 12m | 15D 3m | 15D 12m |
| --- | --- | --- | --- | --- | --- | --- | --- | --- | --- |
| PCS basline | CC | 1 | 0.54 | -0.40 | 0.1 | 0.03 | 0.04 | -0.21 | -0.20 |
|  | P | - | 0.000 | 0.001 | 0.38 | 0.78 | 0.7 | 0.05 | 0.07 |
| PCS 3m | CC |  | 1 | 0.10 | 0.19 | 0.14 | 0.16 | -0.33 | -0.13 |
|  | P |  |  | 0.43 | 0.1 | 0.24 | 0.17 | 0.003 | 0.28 |
| PCS change 0-12m | CC |  |  | 1 | 0.24 | 0.30 | 0.21 | -0.08 | -0.07 |
|  | P |  |  |  | 0.05 | 0.02 | 0.09 | 0.56 | 0.58 |
| PRWE 12m | CC |  |  |  | 1 | 0.83 | 0.74 | -0.45 | -0.41 |
|  | P |  |  |  |  | 0.00 | 0.00 | 0.00 | 0.00 |
| qDASH 12m | CC |  |  |  |  | 1 | 0.66 | -0.63 | -0.63 |
|  | P |  |  |  |  |  | 0.00 | 0.00 | 0.00 |
| VAS for pain 12m | CC |  |  |  |  |  | 1 | -0.28 | -0.37 |
|  | P |  |  |  |  |  |  | 0.01 | 0.001 |
| 15D 3m | CC |  |  |  |  |  |  | 1 | 0.89 |
|  | P |  |  |  |  |  |  |  | 0.00 |
| 15D 12m | CC |  |  |  |  |  |  |  | 1 |
|  | p |  |  |  |  |  |  |  |  |

CC = Pearson’s correlation coefficient

P = p-value, 2-tailed

m = months of follow-up

**Box-plot charts of the PRWE, the Quick-DASH, and VAS for pain by treatment group allocation**


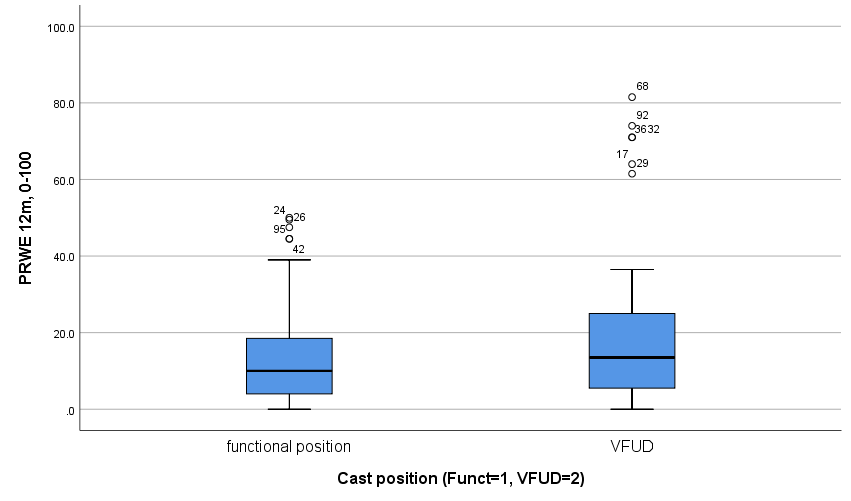


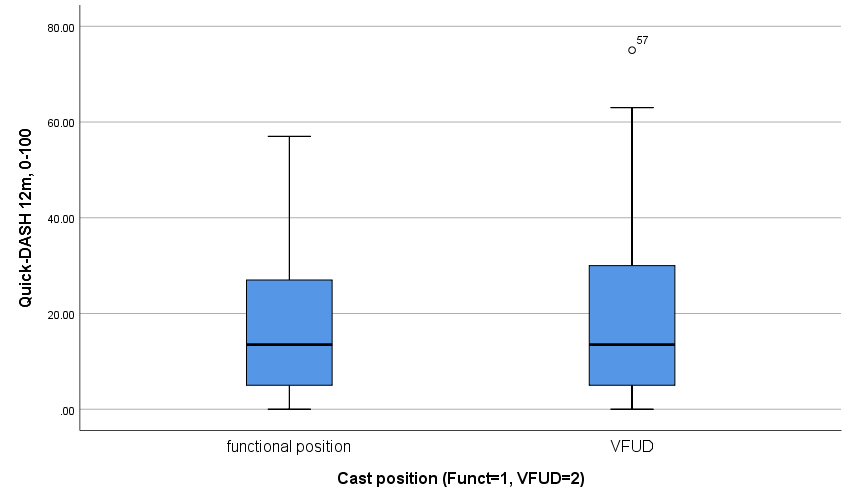


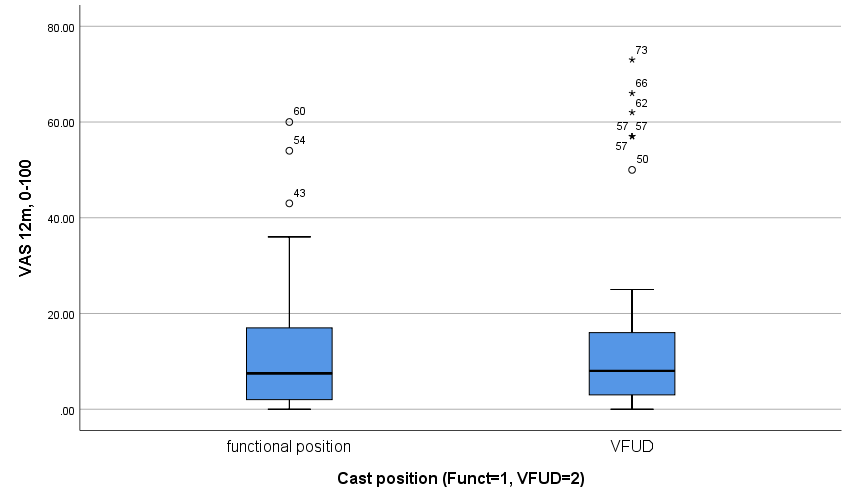

Supplement: S3 Appendix — (DOCX) [file pone.0232153.s004.docx]
