# Supplementary material for: Two casting methods compared in patients with Colles' fracture: A pragmatic, randomized controlled trial
Source: PLoS One. 2020 May 29;15(5):e0232153. doi: 10.1371/journal.pone.0232153 (PMC7259650; doi:10.1371/journal.pone.0232153)
Supplement: S4 Appendix — (DOCX) [file pone.0232153.s005.docx]

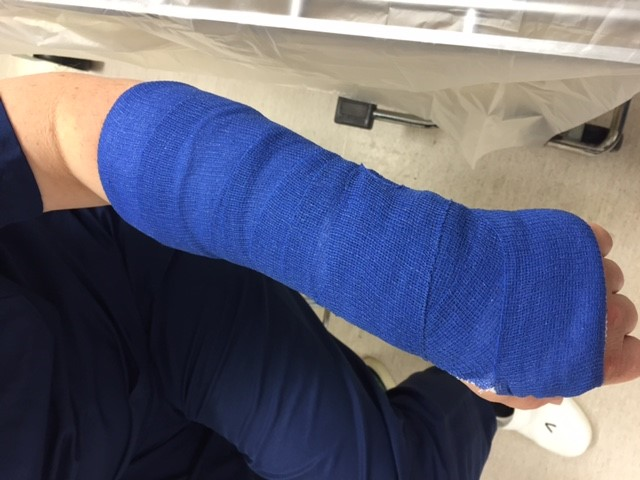


Picture 1. Volar-flexion and ulnar deviation cast, above view


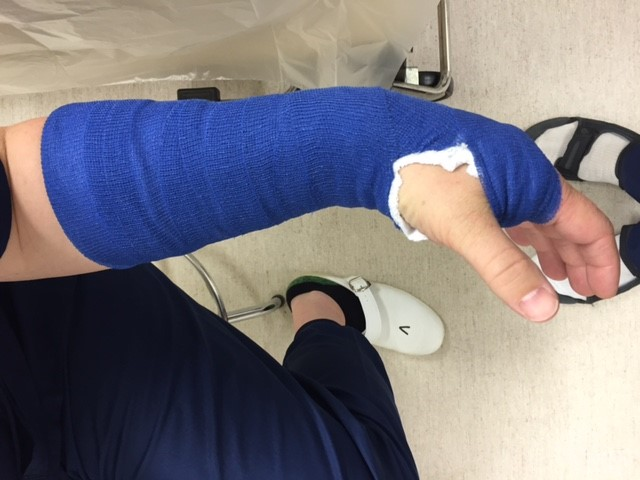


Picture 2. Volar-flexion and ulnar deviation cast, side view


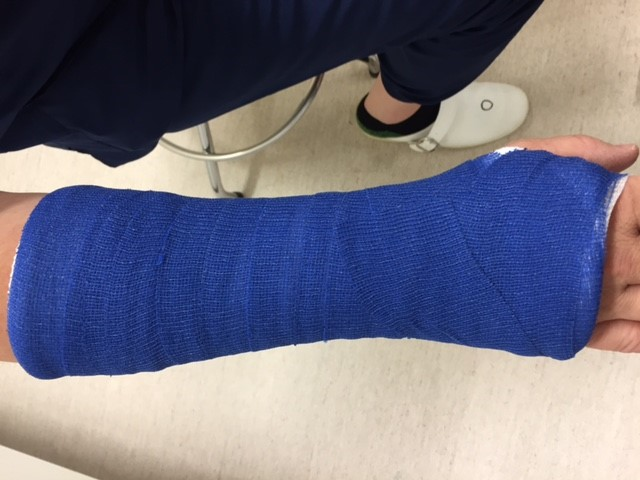


Picture 3. Functional cast, above view


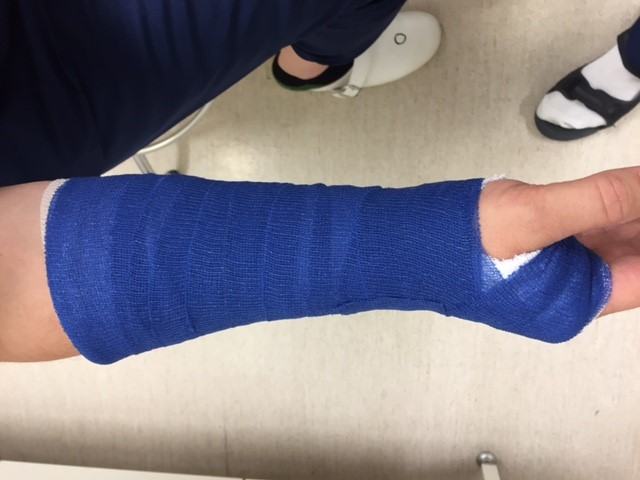


Picture 4. Functional cast, side view
