# Supplementary material for: Two casting methods compared in patients with Colles' fracture: A pragmatic, randomized controlled trial
Source: PLoS One. 2020 May 29;15(5):e0232153. doi: 10.1371/journal.pone.0232153 (PMC7259650; doi:10.1371/journal.pone.0232153)
Supplement: S1 File — (DOC) [file pone.0232153.s007.doc]

**Research plan**

**Nordic Innovative Trial to Evaluate osteoPorotic Fractures (NITEP research group): Pragmatic study on the cast position on non-operatively treated distal radius fractures**

**Participants of the consortium**

**Tampere University Hospital**

Project leader: Ville Mattila, MD, PhD, professor of orthopaedics and traumatology, University of Tampere, the School of Medicine and visiting professor, Department of Clinical Science, Intervention and Technology (CLINTEC), Division of Orthopedics and Biotechnology, Karolinska Institutet, Stockholm, Sweden

Address: Teiskontie 35, PL2000, 33521 Tampere, Finland

Tel: +358-3-311 67674

e-mail: [ville.mattila@uta.fi](mailto:ville.mattila@uta.fi)

Main Researcher: Antti Launonen, MD, PhD, post-doc

Participating researchers (Post-docs):

Vesa Lepola, MD, PhD

Tuomas Huttunen MD, PhD

Scientific advisor: Minna Laitinen, docent

Participating researchers (PhD students):

Bakir Sumrein, MD

Teemu Hevonkorpi, BM

Other participants: Heini Huhtala, statistical consultant

**Central Finland Central Hospital**

Team leader: Toni Luokkala, MD, orthopaedics and traumatology,

Participating researchers (Post-docs):

Aleksi Reito, MD, PhD

**Satakunta Central Hospital**

Team leader Juha Kukkonen, MD, PhD, orthopaedics and traumatology

Participating researcher Ulla Peltoniemi, MD

1. **BACKGROUND**

The impact of degenerative musculoskeletal diseases on individual people and on the society as a whole will increase dramatically in the future across the world due to the increasing age and more sedentary lifestyle of most western populations [1]. Most common osteoporotic fractures are distal radius fracture (DRF), proximal humerus and hip fractures. Although operative treatment of hip fractures is universally accepted, severe discrepancies exist on the treatment policies of the most common upper extremity fractures, distal radius, among the elderly people [2].

Distal radius fractures (DRF) can be categorized to stable or unstable fractures. A stable fracture maintains dorsal tilt, radial height, radial inclination, ulnar variance and carpal malaligment during conservative treatment whilst during cast treatment of an unstable fracture a displacement will eventually occur. In these cases, fracture reduction and anatomical restoration is often acceptable after manipulation and cast application but it will be lost during the treatment. This loss in fracture reduction can occur early (< 1-2 week) or late (at 6 weeks) [3]. Late instability presents a particularly complex problem to clinicians because early radiographs may show good reduction but later fracture might still collapse and lead to functionally disabling malunion. These unstable fractures account for about 30-60% of all DRFs [3-5]. However, since the reason for instability is multifactorial, there is no simple way to predict fracture healing [4-12].

The purpose of conservative treatment is to stabilize the fracture with cast, brace or other external supports where the bone is allowed to heal anatomically. Immobilization should also relieve pain. Several different methods of immobilization have been described [13-18]. Historically most often used method is closed reduction and immobilizing the wrist into slight flexion-ulnar deviation-pronation –position. The flexion -position was first described by Frederic J. Cotton in 1910 [19]. Although the method was commonly used in that era there was growing evidence that a strict wrist flexion could lead to median nerve compression. In 1933 Abbott and Saunders noticed that reduction in flexion increases resistance for dye flow in cadaver carpal tunnels [20]. Later the method was condemned as ‘Cotton-Loder’ position and since then the absolute flexion has been abandoned. Slight flexion-ulnar deviation position was, however, considered to be beneficial for maintaining the fracture reduction during immobilization. It was suggested that in this reduction position the soft tissue envelope around the fracture fragments and the distraction produced by radiocarpal ligaments (ie. ligamentotaxis) could resist the dislocating forces generated over the fracture line. In 1950 Sir John Charnley published his classic and highly influential guideline “The Closed Treatment of Common Fractures” [21]. In his book Charnley described the use of 3-point technique to immobilize the fracture in a slight flexion-ulnar deviation-pronation position. This technique has been widely adopted worldwide by several leading authors in important textbooks of orthopaedics and hand surgery until the recent years.

However, in clinical practice the flexion-ulnar deviation reduction position causes common extensor tendons to be tightened and thereby not allowing proper finger flexion during treatment. This is problematic especially among elderly people in whom degenerative joints are vulnerable to immobilization- induced stiffness. The median nerve compression, the most common complication in DRF, is also been seen n patients with increased carpal tunnel pressure. The pressure has been shown to increase three-fold when changing the neutral cast immobilization position to flexion [22-24].

Other methods of immobilization are functional bracing, immobilization wrist in neutral and slightly extended position as well as in pronation or in supination [13-16]. Cochrane database review regarding conservative treatment of DRF in adults concluded that there is insufficient evidence from randomised trials to determine which method of conservative treatment is the most appropriate when treating most common types of adult DRF [24].

It has been suggested that fracture stability during conservative treatment is not related to cast or immobilization position properties but merely ‘inherent’ characteristics of fracture as well as the patient [4,9-12,24]. Current evidence also indicates that the functional outcome of DRF in elderly people (>65) is not related to anatomic alignment of the fracture [25-30]. Weak evidence supports neutral (radius-metacarpal line 0°) or functional (radius-metacarpal line 20° of extension) reduction [17,18,24]. This practice has been adopted by several countries in their guidelines of DRF treatment. However, the evidence supporting this position is scarce and there are no high quality RCT studies in the English language literature comparing commonly used slight flexion-ulnar deviation -position and functional position.

Functional outome in DRF patients can be affected other than fracture alignment or other fracture spesific factors as well. Pain catastrophizing and fear to use injured limb are shown to increase disability, increased pain and muscle weakness in upper extremity and DRF patients [31-34]. The severity of acute pain, catastrophic thinking and trauma related anxiety has been shown to associate with finger stiffness after DRF [35]. Pain catastrophizing scale (PCS) is one of the most widely used measure of catastrophic thinking related to pain [36]. PCS has been translated into several languages and is widely incorporated in the assessment protocol of pain clinics and rehabilitation centres. However, pain catastophizing as a predictor of funtional outcome has not been previously studied with consevartively treated DRF in elderly people.

1. **OBJECTIVES**

The aims of the present pragmatic collaboration study on the conservative treatment of DRFs are:

1. to compare two cast position (volar flexion, ulnar deviation cast position and functional cast position) by means of functional outcome measured with patient rated wrist evaluation (PRWE) score in persons aged 65 or more.
2. to compare pain, disability, quality of life, grip strength, number of complications and cast changes after DRF treated with these two different cast positions
3. to assess the effect of PCS on the functional outcome of conservatively treated DRFs and the possible interaction between PCS and the functional outcome of DRF treatment with two different cast positions
4. **PRIMARY HYPOTHESES**

Our primary null hypotheses are as follows:

1. functional cast position gives similar functional outcome than currently used flexion-ulnar deviation cast position
2. functional cast position yields to similar results to currently used flexion-ulnar deviation cast position with regard to quality of life and grip strength and also with lower rates of complications and number of cast changes during immobilization when treating DRFs.
3. high PCS does not have an interaction with functional outcome on the patient rated wrist evaluation (PRWE) scale after adjusting for cast position used in the conservative treatment
4. **STUDY DESIGN**

The study is a pragmatic, prospective, randomized, controlled, multi-center trial. It is aimed to compare two different positions of a cast. The two cast positions compared are flexion-ulnar deviation position (widely used in Finland) and functional position used in other Nordic countries.

Primary outcome in this study is PRWE score measured after one and two years. Secondary outcomes measured are disability [Quick-DASH (disabilities of the arm, shoulder and arm)], pain in visual analogue scale (VAS), PCS, quality of life (15-D), complications and number of surgical interventions and cast changes. Subgroup analysis will be performed to find out patient specific features indicating good or worse outcome. PCS is used to assess the presence of possible mental susceptibility possibly influencing to the functional outcome.

1. **RESEARCH METHODS AND MATERIAL**
   1. **PATIENT SELECTION**

The eligible study population will consist of all consecutively (aged 65 or more) treated patients with a DRF identified in the public or referral emergency departments of participating hospitals.

**Following criteria is used in patient selection:**

Inclusion criteria:

- low energy intra- or extra-articular dorsally displaced DRF within 3cm of the radiocarpal joint, diagnosed with lateral and posterior-anterior radiographs in emergency room (ER)
- physician on call (general practician (GP), acute physician, orthopedic resident or orthopaedic consultant) thinks patient could be appropriate for conservative treatment

Exclusion criteria:

- Operative treatment
- Refuse to participate the study
- Open fracture more than Gustilo 1 gradus
- Age 64 or less
- Chauffeure’s or Barton´s fracture
- Smith´s fracture (volar angulation of the fracture)
- Does not understand written and spoken guidance in local languages
- Pathological fracture or (previous) fracture in the same wrist, antebrachium or elbow
  1. **RANDOMISATION**

All patients will be randomized before closed reduction of displaced fractures and immobilization without reduction of non-displaced fractures to either functional cast or flexion-ulnar deviation cast for 5 weeks. Patients will be randomized using a random number matrix in block allocation fashion. The blocks will be stratified by the known associations; age, centre and intra or extra-articular fracture line. The treatment allocations from the matrix will be sealed in envelopes, which are located in the ER. After the patient´s enrollment has been confirmed and informed consent is signed, a medical orderly specialized in casting will open the envelope and make the cast as allocated. The medical orderlies responsible for the casting will not participate in other ways in the study.

The research coordinator will monitor the study flow. An independent monitoring committee has been established.

- 1. **INTERVENTION**

DRFs are in everyday life treated by health care center physicians, experienced acute physicians, and trauma surgeons, thereby; we do not see any reason to limit the number of on call physicians or education or experience of on-call physician in this pragmatic trial.

Closed reduction is done under local anaesthesia by injecting Lidocaine 1% into fracture area. The technique of closed reduction is not limited to any specific technique. Fluoroscopy can be used when performing closed reduction if it is available in the participating center. Cast reduction position examples are delivered to participating centers to assure correct position of casting. After closed reduction, additional radiographs will be taken to confirm the reduction of the fracture. After physician on-call has accepted the fracture to be treated conservatively, a dorsal splint for 5 weeks is used. Non-displaced fractures are immobilized according to randomization without reduction.

After the allocation and preparing the cast, the patient is asked to fill the PCS questionnaire.

- 1. **FOLLOW-UP**

Follow-up visits are being conducted in primary health care centers as seen necessary by the physician on-call. They are arranged by the regional guidelines, at 1 and 2 weeks with radiographs. Due to the pragmatic nature of the trial we will not set any angulation degrees or shortening limits indicating operative treatment if reduction is lost during the follow-up. Whether the achieved angulation and/or possibly restored shortening is lost during the follow-up, the decision to consult local orthopedist or hand surgeon is left to the discretion of treating physician. Moreover, if patient has been referred for operative treatment from the primary care by the physician on-call but conservative treatment is chosen in the referral center ,the patient is still eligible for the study. Follow-up of these patients is conducted in the primary care or hospital based on local protocols. Since patients will be randomized in blocks by the hospital, we will assume that randomization will take care of the possible differences between centers.

**Figure**: Flow chart of the trial.

**Allocation + initial assessment**

Assessed for eligibility

>65 years old

Physician on-call thinks that patient is suitable for non-operative treatment

Fracture reduction if necessary

GP´s visit and x-ray prior to cast removal

♦ Mailed Questionnaires, no physician´s visit

♦ PRWE, QuickDash, VAS, 15-D, PCS

**1 +2 week Follow-Up**

Randomization by the ER personnel

**Enrollment**

**5 w follow-up**

**1+2 y Follow-Up**

Allocation to volar flexion & ulnar deviation cast and PCS

GP´s visit + x-ray if seen necessary by the physician on-call

♦ Physician´s visit at study hospital, x-ray

♦ PRWE, QuickDash, VAS, PCS, 15-D ROM, grip

**3 m Follow-Up**

GP´s visit and x-ray prior to cast removal

♦ Mailed questionnaires, no physician´s visit

♦  PRWE, QuickDash, VAS, 15-D, PCS

♦ Physician´s visit at study hospital, x-ray

♦ PRWE, QuickDash, VAS, PCS, 15-D ROM, grip

Excluded

Direct operative treatment

Refuse to participate the study

Open fracture more than Gustilo 1 gradus

Age 64 or less

Chauffeure’s or Barton´s fracture

Smith´s fracture (volar angulation of the fracture)

Does not understand written and spoken guidance in local languages

Pathological fracture or previous fracture in the same wrist, antebrachium or elbow

Pathological fracture or previous fracture in the same upper extremity

Allocation to functional cast and PCS

GP´s visit + x-ray if seen necessary by the physician on-call

Cast immobilization time will be 5 weeks according to current guidelines in Finland. The final physician’s visit will be at the time of the cast removal. All patients have an x-ray prior to cast removal. After cast removal, the decision to refer patient for physiotherapist will be left to discretion of the treating physician.

After three months the patient will visit orthopedic outpatient clinic in the hospital where the treatment was initially started (Table). This visit is part of the study protocol. During the visit a direct lateral and anteroposterior radiographs will be taken. PRWE, PCS, Quick-DASH, 15-D and pain in VAS will be assessed. The number of cast changes during treatment is queried from the patient and the number of changes is recorded.

The 1- and 2-year **follow-ups are executed with postal questionnaires in order to reduce patients’ discomfort and aiming to as easy-as possible handling of the follow-up data. Controls include** PRWE, VAS (for level of pain), PCS, Quick-DASH and 15-D.

At the 1 and 2 year follow-up complications will be recorded from patient records.

|  | **Medical history** | **Radiograph** | **PRWE** | **PCS** | **Pain** | **Grip** | **Quick-DASH** | **15-D** | **VAS** |
| --- | --- | --- | --- | --- | --- | --- | --- | --- | --- |
| **Baseline** | x | X |  | x |  |  |  |  |  |
| **1-2 weeks** | (x) | (x) |  |  |  |  |  |  |  |
| **5-6 weeks** | (x) | X |  |  |  |  |  |  |  |
| **3 months** | x | X | x | x | x | x | x | x | x |
| **1 year** |  |  | x | x | x |  | x | x | x |
| **2 years** |  |  | x | x | x |  | x | x | x |

(x) = taken if required by the treating GP

**Table**: Assessments and procedures of the trial.

- 1. **POWER ANALYSIS**

In this trial, a validated wrist specific PRWE-score is used as the primary outcome measure. Just recently Wallenkamp and coworkers published a report, where minimal clinically important difference in PRWE was set at 11 points with SD of 14 [37]. Based on power calculations (Cl 95%, power 0.95, SD 14) required sample size per group is 40 patients. Assuming 30% of drop-out due to possible surgical interventions during cast treatment, group size would be 52 per group (total 104). In case of patient cross-over, patients will be analyzed according to the intention-to-treat principle.

- 1. **ANALYSIS OF THE MATERIAL**

Each patient will get an initial trial identification number (TIN) which is matched with actual patient identification (ID). **The matching key is stored in study nurse’s office in Tampere University Hospital. Patients are handled only with TIN during follow-up period and analyzing the of the material. Results are given only in general level with mean values thus individual patient’s information is not able to be extracted. The electronical registry contains x-rays and it is located in hospital’s secure server. The research data will be deleted 15 years after the end of the study.**

- 1. **STATISTICAL ANALYSIS**

Continuous skewed variables between the groups were compared by the Mann-Whitney *U* test and t-test is used when variables are unskewed. Results are presented with 95% confidence intervals. Two-way-tables with the chi-square test will be used for dichotomous variables. In subgroup analysis the effect of age, sex, fracture group, smoking, and other diseases will be evaluated against the scores and overall quality of life after fracture.

Analysis of covariance will be used to assess the effect of pain catastrophizing score on the outcome of cast treatment. PRWE will be used as a dependent variable, cast as independent and pain catastrophizing score as covariate.

The effect of cast position and PRWE is investigated also in the multivariate manner. Multivariable analysis is performed with linear regression analysis since the outcome variable PRWE is normally distributed. Main variable of interest included is the cast and age, sex, fracture group, smoking and other diseases are used as confounding variables.

- 1. **ETHICAL ISSUES**

The ethical approval of this study is applied with this research plan.

1. **IMPLEMENTATION**
   1. **CONTINGENCY PLAN**

In Finnish population aged 60 years or more the incidence of DRF can be estimated to be 126-190 per 100 000 person years [38,39]. We need to recruit more than 100 patients as suggested by the power calculation. Time for accrual in a single center is estimated to be too long; therefore to be able to enroll sufficient number of patients we are conducting a multicenter study. This study will be carried in Tampere University hospital, in Central Finland Central hospital and Satakunta Central hospital. Tampere has a catchment area with a population of 300 000 people who in case of an emergency are referred to emergency department located in the Tampere University hospital. The two latters have a catchment area with a population of 100 000 to 150 000 people who are referred to Central Finland and Satakunta Central hospitals in case of an emergency. With the incidence indicated above, we’ll identify 650-950 DRFs among elderly patients aged 60 or more within one year. Therefore we assume to finish recruiting study patients within one year.

We have homogeneity between centers with solid trial designs, data and project management including monitoring. A site personnel training has been carried out. We have been assisted by local research centers. Stability of the trial has been maintained with regular mails where researchers have shared concerns, problems and successes. Regular annual meetings (twice a year, previous on Jan 15, 2016 in Stockholm) will motivate the research team and maintain stable quality in treating. Meetings also include presentations of future studies and trials, methodology and scientific meanings will be debated. Homepage of the research team including current trial (www.nitep.eu) is under coding.

1. **Expected results and impact**
2. We expect to observe better functional outcome after functional cast treatment compared to flexion -ulnar deviation retention cast
3. We expect to observe better grip strength, quality of life, less cast changes and smaller pain in VAS with functional cast compared to flexion-ulnar deviation retention cast
4. Functional outcome is assumed to correlate with PCS regardless of treatment modality
5. After publishing these results we aim to support the creation and dissemination of trustworthy guidelines by health authorities and professional organizations.
6. We expect that after demonstrating clinical results (our RCT’s) and supporting the creation and dissemination of trustworthy guidelines treatment policy of DRF’s will be substantially changed, to reflect best current evidence.

References

1. Woolf AD, Pfleger B. Burden of major musculoskeletal conditions. Bull World Health Organ. 2003;81: 646-656.

2. Larsen CF, Lauritsen J. Epidemiology of acute wrist trauma. Int J Epidemiol. 1993;22: 911-916.

3. Leone J, Bhandari M, Adili A, McKenzie S, Moro JK, Dunlop RB. Predictors of early and late instability following conservative treatment of extra-articular distal radius fractures. Arch Orthop Trauma Surg. 2004;124: 38-41.

4. Wadsten MA, Sayed-Noor AS, Englund E, Buttazzoni GG, Sjoden GO. Cortical comminution in distal radial fractures can predict the radiological outcome: a cohort multicentre study. Bone Joint J. 2014;96-B: 978-983.

5. Altissimi M, Mancini GB, Azzara A, Ciaffoloni E. Early and late displacement of fractures of the distal radius. The prediction of instability. Int Orthop. 1994;18: 61-65.

6. Lafontaine M, Hardy D, Delince P. Stability assessment of distal radius fractures. Injury. 1989;20: 208-210.

7. Hove LM, Solheim E, Skjeie R, Sorensen FK. Prediction of secondary displacement in Colles' fracture. J Hand Surg Br. 1994;19: 731-736.

8. Abbaszadegan H, Jonsson U, von Sivers K. Prediction of instability of Colles' fractures. Acta Orthop Scand. 1989;60: 646-650.

9. Nesbitt KS, Failla JM, Les C. Assessment of instability factors in adult distal radius fractures. J Hand Surg Am. 2004;29: 1128-1138.

10. Mackenney PJ, McQueen MM, Elton R. Prediction of instability in distal radial fractures. J Bone Joint Surg Am. 2006;88: 1944-1951.

11. Makhni EC, Taghinia A, Ewald T, Zurakowski D, Day CS. Comminution of the dorsal metaphysis and its effects on the radiographic outcomes of distal radius fractures. J Hand Surg Eur Vol. 2010;35: 652-658.

12. Tahririan MA, Javdan M, Nouraei MH, Dehghani M. Evaluation of instability factors in distal radius fractures. J Res Med Sci. 2013;18: 892-896.

13. Blatter G, Papp P, Magerl F. A comparison of 2 methods of plastic cast fixation in treatment of loco classico radius fracture. A prospective, randomized study. Unfallchirurg. 1994;97: 534-540.

14. Sarmiento A, Zagorski JB, Sinclair WF. Functional bracing of Colles' fractures: a prospective study of immobilization in supination vs. pronation. Clin Orthop Relat Res. 1980;(146): 175-183.

15. Wilson C, Venner RM. Colles' fracture. Immobilisation in pronation or supination? J R Coll Surg Edinb. 1984;29: 109-111.

16. Wahlstrom O. Treatment of Colles' fracture. A prospective comparison of three different positions of immobilization. Acta Orthop Scand. 1982;53: 225-228.

17. Gupta A. The treatment of Colles' fracture. Immobilisation with the wrist dorsiflexed. J Bone Joint Surg Br. 1991;73: 312-315.

18. van der Linden W, Ericson R. Colles' fracture. How should its displacement be measured and how should it be immobilized? J Bone Joint Surg Am. 1981;63: 1285-1288.

19. Cotton F.  *Dislocations and Joint Fractures*. 1st ed. Philadelphia and London: W.B. Saunders Company; 1910.

20. Abbott L, Saunders J. Injuries of the median nerve in fractures of the lower end of the radius. Surg Gynecol Obstet. 1933;57: 507.

21. Charnley J. *The Closed Treatment of Common Fractures*. 1st ed: The Williams and Wilkins Company; 1950.

22. Gelberman RH, Szabo RM, Mortensen WW. Carpal tunnel pressures and wrist position in patients with colles' fractures. J Trauma. 1984;24: 747-749.

23. Dyer G, Lozano-Calderon S, Gannon C, Baratz M, Ring D. Predictors of acute carpal tunnel syndrome associated with fracture of the distal radius. J Hand Surg Am. 2008;33: 1309-1313.

24. Handoll HH, Madhok R. Conservative interventions for treating distal radial fractures in adults. Cochrane Database Syst Rev. 2003;(2): CD000314.

25. Arora R, Lutz M, Deml C, Krappinger D, Haug L, Gabl M. A prospective randomized trial comparing nonoperative treatment with volar locking plate fixation for displaced and unstable distal radial fractures in patients sixty-five years of age and older. J Bone Joint Surg Am. 2011;93: 2146-2153.

26. Grewal R, MacDermid JC. The risk of adverse outcomes in extra-articular distal radius fractures is increased with malalignment in patients of all ages but mitigated in older patients. J Hand Surg Am. 2007;32: 962-970.

27. Abbaszadegan H, Jonsson U. External fixation or plaster cast for severely displaced Colles' fractures? Prospective 1-year study of 46 patients. Acta Orthop Scand. 1990;61: 528-530.

28. Azzopardi T, Ehrendorfer S, Coulton T, Abela M. Unstable extra-articular fractures of the distal radius: a prospective, randomised study of immobilisation in a cast versus supplementary percutaneous pinning. J Bone Joint Surg Br. 2005;87: 837-840.

29. McQueen MM, Hajducka C, Court-Brown CM. Redisplaced unstable fractures of the distal radius: a prospective randomised comparison of four methods of treatment. J Bone Joint Surg Br. 1996;78: 404-409.

30. Stein H, Volpin G, Horesh Z, Hoerer D. Cast or external fixation for fracture of the distal radius. A prospective study of 126 cases. Acta Orthop Scand. 1990;61: 453-456.

31. Linton SJ, Nicholas MK, MacDonald S, Boersma K, Bergbom S, Maher C, et al. The role of depression and catastrophizing in musculoskeletal pain. Eur J Pain. 2011;15: 416-422.

32. Jelicic M, Kempen GI. Do psychological factors influence pain following a fracture of the extremities? Injury. 1999;30: 323-325.

33. Roh YH, Lee BK, Noh JH, Oh JH, Gong HS, Baek GH. Effect of anxiety and catastrophic pain ideation on early recovery after surgery for distal radius fractures. J Hand Surg Am. 2014;39: 2258-64.e2.

34. Das De S, Vranceanu AM, Ring DC. Contribution of kinesophobia and catastrophic thinking to upper-extremity-specific disability. J Bone Joint Surg Am. 2013;95: 76-81.

35. Teunis T, Bot AG, Thornton ER, Ring D. Catastrophic Thinking Is Associated With Finger Stiffness After Distal Radius Fracture Surgery. J Orthop Trauma. 2015;29: e414-20.

36. Sullivan MJL, Bishop SR, Pivik J. The Pain Catastrophizing Scale: Development and validation. Psychol Assess. 1995;7: 524-532.

37. Walenkamp MM, de Muinck Keizer RJ, Goslings JC, Vos LM, Rosenwasser MP, Schep NW. The Minimum Clinically Important Difference of the Patient-rated Wrist Evaluation Score for Patients With Distal Radius Fractures. Clin Orthop Relat Res. 2015;473: 3235-3241.

38. Koski AM, Patala A, Patala E, Sund R. Incidence of osteoporotic fractures in elderly women and men in Finland during 2005-2006: a population-based study. Scand J Surg. 2014;103: 215-221.

39. Flinkkila T, Sirnio K, Hippi M, Hartonen S, Ruuhela R, Ohtonen P, et al. Epidemiology and seasonal variation of distal radius fractures in Oulu, Finland. Osteoporos Int. 2011;22: 2307-2312.
