# Supplementary material for: Two casting methods compared in patients with Colles' fracture: A pragmatic, randomized controlled trial
Source: PLoS One. 2020 May 29;15(5):e0232153. doi: 10.1371/journal.pone.0232153 (PMC7259650; doi:10.1371/journal.pone.0232153)
Supplement: Attachment — Submitted filename: Response to Reviewers.docx [file pone.0232153.s008.docx]

Rebuttal letter

We would like to take this opportunity to thank the Reviewers for their constructive criticism of our manuscript. We have strived to revise the manuscript in accordance with the valuable criticism received from the Reviewers. We feel that the quality of our manuscript has improved as a result.

The comments of the Reviewers are followed by our answers and a description of the corrections/changes that we have made to the manuscript (marked in *italics*).

Reviewers' comments:

1. Is the manuscript technically sound, and do the data support the conclusions?

The manuscript must describe a technically sound piece of scientific research with data that supports the conclusions. Experiments must have been conducted rigorously, with appropriate controls, replication, and sample sizes. The conclusions must be drawn appropriately based on the data presented.

Reviewer #1: Yes

Reviewer #2: Yes

2. Has the statistical analysis been performed appropriately and rigorously?

Reviewer #1: Yes

Reviewer #2: Yes

3. Have the authors made all data underlying the findings in their manuscript fully available?

The PLOS Data policy requires authors to make all data underlying the findings described in their manuscript fully available without restriction, with rare exception (please refer to the Data Availability Statement in the manuscript PDF file). The data should be provided as part of the manuscript or its supporting information, or deposited to a public repository. For example, in addition to summary statistics, the data points behind means, medians and variance measures should be available. If there are restrictions on publicly sharing data—e.g. participant privacy or use of data from a third party—those must be specified.

Reviewer #1: Yes

Reviewer #2: No

*We have now verified the policies of our Regional Ethical Committee on data sharing. In order to ensure the anonymization of the dat. Hence, the exact day and month of the recruitment has been removed and study centers are only referred to as numbers one, two, and three. Due to the common nature of distal radius fracture, we feel these changes enable the required anonymization of the data. The data will be submitted in the supplementary file alongside the manuscript.*

4. Is the manuscript presented in an intelligible fashion and written in standard English?

PLOS ONE does not copyedit accepted manuscripts, so the language in submitted articles must be clear, correct, and unambiguous. Any typographical or grammatical errors should be corrected at revision, so please note any specific errors here.

Reviewer #1: Yes

Reviewer #2: No

5. Review Comments to the Author

Reviewer #1: well done study statistical methodology sound addresses a contemporary question now that so many of these fractures are treated operatively. This reviewer has little critique with your overall methodology nor conclusions.

*The authors thank the reviewer for the positive comments*.

Reviewer #2: This investigation is well and completely reported and discussed. The English language is poor, even unclear, in parts (e.g., section ‘PCS’ in the appendix) and needs to be revised by a native speaker or other competent person. I have only minor comments and suggestions:

*The authors thank the reviewer for the positive comments. The material in the supplementary files has now been revised by a native speaker of English.*

1. Please state the randomisation block size. According to the stated stratification factors, there seem to have been 2x3x2 = 12 strata, which for planned 57 patients per group would seem to lead to very numbers in each stratum**.**

*In the randomization process, we had two stratification variables, age and extra- or intra-articular fracture line, with two blocks in each variable. Thus, there were 4 stratification factors (lines 116-7).*

2. The statistics software used for data analysis should be stated.

*The statistical software used for the data analysis was SPSS v. 25 (lines 147-8).*

3. Detail of the multiple imputation procedure used should be given in the appendix.

*The variables used for the multiple imputation were* *age, sex, cast position (treatment allocation), the PRWE at 3 and 12 months, the PCS at 3 and 12 months, the VAS for pain at 3 and 12 months, and the Quick-DASH at 3 and 12 months. This has now been explained in more detail in appendix S4.*

4. Please state which outcome variables were collected at baseline, and the time-point of the outcome. In Table 2, only PCS is given at baseline. Further, which ‘difference’ is meant in the sentence beginning ‘The primary outcome measure was the difference…’, p.5? I understand that the PRWE was not measured at baseline, so presumably you mean the difference between the treatment groups? Generally, an ‘outcome’ should be defined on the individual patient.

*The mean difference between study groups at 12 months was the primary outcome. This has now been stated more clearly (lines 126-7).*

5. I assumed that all localised outcome measures refer to the injured wrist, but on p.8 we are informed about the correlation with grip strength of the uninjured limb – please clarify.

*The reviewer is indeed correct that we have reported the correlation with the grip strength of the uninjured limb to the PRWE score in order to find an association, if any, between the physical strength of the upper arms and the primary outcome.*

6**.** When were the radiograph examinations and assessments performed, and were the assessors blinded to treatment group?

*The radiograph examinations were performed before and after the closed reduction, 1, 2, and 5 weeks after fracture. The radiographic assessments were performed by authors who were blinded to the treatment allocation at the time of assessments (lines 133-4).*

7. I suggest displaying the distribution of primary (and possibly important secondary) outcome values graphically, for instance using box-plots or scatter plots comparing the treatment groups.

*The authors fully agree with the usefulness of the graphical reporting of the main results. We have now added a box-plot graph that contains the results of the PRWE, the Quick-DASH, and VAS for pain in appendix S4.*

8. Were subgroup analyses per site performed and if so, were any differences found? In such a surgery study, where physician-specific effects may be expected, it would be important to look for site subgroup differences in the primary treatment comparison.

*In each study center, recruitment to the study and the closed reduction of the fracture was performed by various on-call physicians in the emergency departments. Therefore, each physician only recruited and treated a few patients in the study. The subgroup analysis of radiograph parameters for the internal validation of the allocated treatment showed no difference between study centers.*

9. On p.13 (discussion) a cast change rate per patient of 260% is referred to – does this mean multiple changes in some patients?

*This rate of cast changes has been referred to in a paper published by Anzarut et a.l (ref 26 in the manuscript) who reported in Table 1 of the article the mean of 2.6 and the median of 2 cast changes in the study population. However, we are not confident in this reported number and we have therefore changed the sentence: “Studies investigating cast immobilization methods have not routinely reported on cast changes, but studies have reported various rates of cast changes” (lines 270-1).*
